# MRI-Based Regional Muscle Use during Hamstring Strengthening Exercises in Elite Soccer Players

**DOI:** 10.1371/journal.pone.0161356

**Published:** 2016-09-01

**Authors:** Alberto Mendez-Villanueva, Luis Suarez-Arrones, Gil Rodas, Rodrigo Fernandez-Gonzalo, Per Tesch, Richard Linnehan, Richard Kreider, Valter Di Salvo

**Affiliations:** 1 Football Performance & Science Department, ASPIRE Academy, Doha, Qatar; 2 Sports Department, Pablo de Olavide University, Sevilla, Spain; 3 Medical Department, Futbol Club Barcelona, Barcelona, Spain; 4 Department of Physiology and Pharmacology, Karolinska Institutet, Stockholm, Sweden; 5 National Aeronautics and Space Administration, Johnson Space Center, Houston, Texas, United States of America; 6 Department of Health and Kinesiology, Texas A&M University, College Station, Texas, United States of America; 7 Department of Movement, Human and Health Sciences, University of Rome “Foro Italico”, Rome, Italy; Universidad Europea de Madrid, SPAIN

## Abstract

The present study examined site-specific hamstring muscles use with functional magnetic resonance imaging (MRI) in elite soccer players during strength training. Thirty-six players were randomized into four groups, each performing either Nordic hamstring, flywheel leg-curl, Russian belt or the hip-extension conic-pulley exercise. The transverse relaxation time (T_2_) shift from pre- to post-MRI were calculated for the biceps femoris long (BFl) and short (BFs) heads, semitendinosus (ST) and semimembranosus (SM) muscles at proximal, middle and distal areas of the muscle length. T_2_ values increased substantially after flywheel leg-curl in all regions of the BFl (from 9±8 to 16±8%), BFs (41±6–71±11%), and ST (60±1–69±7%). Nordic hamstring induced a substantial T_2_ increase in all regions of the BFs (13±8–16±5%) and ST (15±7–17±5%). T_2_ values after the Russian belt deadlift substantially increased in all regions of the BFl (6±4–7±5%), ST (8±3–11±2%), SM (6±4–10±4%), and proximal and distal regions of BFs (6±6–8±5%). T_2_ values substantially increased after hip-extension conic-pulley only in proximal and middle regions of BFl (11±5–7±5%) and ST (7±3–12±4%). The relevance of such MRI-based inter- and intra-muscle use in designing more effective resistance training for improving hamstring function and preventing hamstring injuries in elite soccer players should be explored with more mechanistic studies.

## Introduction

Hamstring muscle tears are the most common muscle injuries in male football players, and are associated with significant time loss and high financial costs for the player and clubs [1, 2]. Thus, adequate prevention and rehabilitation processes are of major importance in this cohort group.

The predominant hamstring injury mechanisms in football occur during high-speed running and/or acceleration efforts [1, 3], or during movements with large joint excursions (i.e., stretching-type injury) such as high-kicking, split positions and glide tackling [4]. Hamstring injuries in football most commonly involve the proximal muscle-tendon unit junction (MTJ) of the BFl, accounting for approximately 60–85% of all hamstrings injuries [4–7].

The occurrence of hamstring muscle strains in football is generally believed to be related with the presence of repetitive high force eccentric actions [8], such as the ones observed during high-speed running [9], where the lengthening demands placed on the muscle could exceed the mechanical limits of the tissue. Increasing the eccentric strength of the hamstring muscles has therefore been proposed as a method to prevent hamstring injuries [8].

Studies have reported that changes in morphology (e.g., anatomical cross-sectional area, muscle thickness) and architecture (e.g., fascicle length, pennation angles) in response to resistance training occurs non-uniformly along the length of the muscle [10, 11]. This non-uniform muscle adaptation to resistance exercise is particular true for eccentric training [12]. The non-uniform change in muscle morphology and architecture after a training intervention has been attributed to the region-specific muscle activation assessed by the transverse relaxation time (T_2_) of functional magnetic resonance images (fMRI) during the training session [10, 11].

Several studies have reported inhomogeneous muscle use within [13–15] and among [16, 17] the four muscles of the hamstring complex during resistance exercises commonly employed in the prevention and rehabilitation of hamstring muscle strains in football players. For example, Mendiguchia et al. (2013b) examined 15 different MR sections during the forward lunge and the eccentric leg curl showing that the leg curl preferentially targeted the ST, whereas the lunge preferentially targeted the proximal portion of the BFl [15]. The same authors [14] reported a non-uniform MR-derived muscle use in the Nordic hamstring exercise, with a preferential use of the ST and BF. Kubota et al. (2007) found a greater usage of the proximal and middle regions than the distal region of the ST following an eccentric prone leg curl exercise. Overall, those between exercise region-specific muscle use differences suggest that specific morphological and architectural adaptations might be elicited with the combination of different hamstring exercises [10]. However, those previous studies did not investigate muscle use in elite, professional soccer players performing contemporary strengthening exercises currently employed to enhance hamstring muscle function and/or prevent and rehabilitate hamstring muscle tears. The high injury rate for hamstring muscles injuries in contemporary football players [18], and the fact that hip extensors and knee flexors eccentric strength [8] have been considered a risk factor for hamstring strain, support the rationale that hamstrings strength should be considered an important component of any training program for football players. Determining the region-specific muscle use of commonly used prophylactic and therapeutic exercises in elite soccer players is relevant for designing the type of resistance training that may be most effective for enhancing hamstring muscle quality and hence preventing initial or recurrent hamstring injuries within this cohort. Specifically, resistance training programs aiming at injury prevention would ideally incorporate regional muscle use aspects that are most similar to the conditions associated with injury, such that the athlete can optimize the gains in functional strength and minimize the risk of future injury. Therefore, the propose of the present study was to examine regional differences in exercise-induced shifts in T_2_ in selected eccentric-biased strengthening exercises commonly used to prevent and/or rehabilitate hamstring injuries in elite soccer players.

## Methods

### Participants

**Fig 1:**
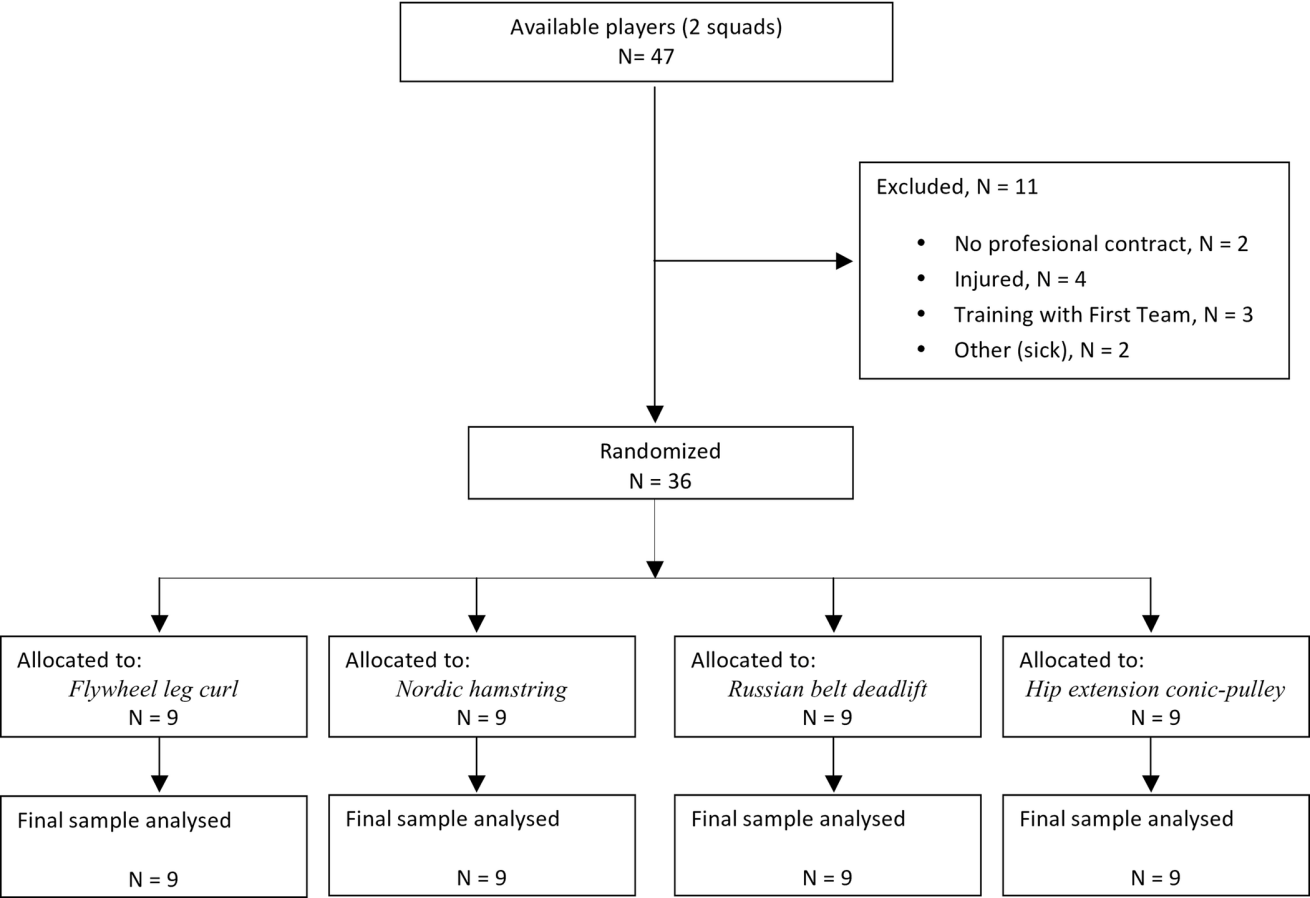
Flow chart showing the selecction of a study sample.

The study examined 36 healthy elite, male professional football players (age 18.4 ± 1.6 yr; height 177.5 ± 1.0 cm; weight 71.0 ± 6.2 kg) belonging to two of the reserve squads of a Spanish La Liga Club. In the last 10 seasons, the first team squad has been ranked among the top 6, being 3 season ranked as the top team, in the official UEFA ranking (www.uefa.com/memberassociations/uefarankings/club/seasonclub/index.html). All the players trained ~ 8 hours of soccer training plus 1 or 2 competitive games per week. To be eligible for the study, players were required to meet the following criteria (Fig 1): (i) to have a current professional contract with the one of the reserve squads of the club; (ii) to be injury free at the moment of the study; and (iii) not being training with the First Team. The purpose and experimental protocol was explained to the players and written informed consent was obtained from the players (or tutor for players under 18). The present study was approved by the local Institutional Research Ethics Committee (i.e., Qatar Antidoping Lab), and conformed to the recommendations of the Declaration of Helsinki.

### Experimental design

The present study used a repeated-measures research design to investigate the regional-specific differences of fMRI measurements in the hamstring muscles, before and after four commonly employed exercises to strengthening and rehab hamstring muscles in football players: flywheel leg curl, Nordic hamstring, Russian belt deadlift and one leg hip-extension conic-pulley. The fMRI included all the thigh in 12 images, and the analysis was performed at different length sections of muscle´s biceps femoris long head (BFl), biceps femoris short head (BFs), semitendinosus (ST) and semimembranosus (SM) before and immediately following 4 exercises [17].

On the experiment day and 30 min before the exercise, players underwent fMRI of both thighs at rest. Then, players performed a 15 min standardized warm up that included: jogging, lower limb joint mobility exercises, dynamic and active stretching exercises, running technique drills, and bodyweight squat and frontal lunge exercises. This was followed by one submaximal set of 8 repetitions of the hamstring exercise the player had to perform later. Lastly, players performed the strength training protocol. Immediately after finishing the training session (within 3–5 min), the subjects underwent fMRI of both thighs.

### Exercise protocol

Players were randomly assigned to one of the four groups/exercises. That is, 9 players performed each exercise. Training session consisted of 4 sets of 8 repetitions. There was a 2-min rest between each set.

#### Flywheel leg curl

A non-gravity dependent supine head-down flywheel leg curl machine was used (YoYo Technology AB, Stockholm, Sweden). Players performed unilateral knee flexor actions using the dominant leg (with hip fixed at 140° angle and the contra-lateral leg rested firmly on the floor), accelerating the flywheel (Inertia 2; 0.07208 kg/m^2^ moment inertia) by concentric (CON) hamstring action and subsequently decelerating it with eccentric (ECC) action of the same muscle group. Players were instructed to apply maximal effort from a straight knee position until full knee flexion, then start braking upon passing the 90° position on the way back and continue braking with maximal effort until the knees were straight (without reaching a full extension). Once the flywheel had come to a stop, the next cycle was initiated [3, 17, 19] (S1 Video).

#### Nordic hamstring

Player started in a kneeling position, with his torso from the knees upward held rigid and straight. A researcher applied pressure to the player’s heels/lower legs to ensure that the feet stay in contact with the ground throughout the movement. The player then attempted to resist a forward-falling motion for as long as possible using their hamstring, and to try keeping tension in their hamstrings even after they have to “let go”. Players used their arms and hands to buffer the fall, let the chest touch the surface, and immediately get back to the starting position [17, 20, 21] (S2 Video).

#### Russian belt deadlift

Players performed the exercise positioned above a slightly inclined platform (~ 45°) and the Russian belt rolled into the area just above their knee. Similar to a deadlift exercise, and with a pelvis anteversion performed just before starting the exercise, players leaned forward (i.e., hip flexion) during the eccentric phase to try to touch the floor with their hands. Afterwards, players initiated hip extension during the concentric phase to return to the starting position [17] (S3 Video).

#### Hip extension conic-pulley

A non-gravity dependent inertial conic-pulley device was used (VersaPulley portable; VersaClimber UK). Players performed the exercise lying supine on a mat with the strap placed around the ankle. The CON hip extension (and slight knee extension) is done during descending phase accelerating the pulley (Inertia 10.8; 0.21964 kg/m^2^ moment inertia), and ECC hip extension to counteract hip flexion (and slight knee flexion) decelerating the pulley is done during ascending phase. Core muscle activation was emphasized during the exercise, and the free leg was blocked by a coach to not rise [17] (S4 Video).

### Imagine technique

All fMRI measurements of the thigh were performed using a 3 T whole-body imager with surface phased-array coils (Siemens, Erlagen, Germany) as described elsewhere [17]. For the fMRI scans, the subjects were positioned supine with their knees extended. All the scans were performed 30 min before and within 3–5 min after the exercise. Once the subject was positioned inside the magnet, the thighs of both legs were kept parallel to the fMRI table, and a custom-made foot-restrain device was used to standardize and fix limb position and to avoid any compression of thigh muscles. Subjects were supine on the MR-gurney with thighs covered with one 32- and 2 flexible 4-channel coils, respectively in the proximal and distal segments. 12 cross-sectional images of the thigh of both legs were obtained, starting at the very distal margin of the ischial tuberosity, and using the following scan sequences: (a) axial fat-suppressed proton density, TR 3000 ms, TE 30–33, echo train 4, slice thickness 3.5 mm, gap 28 mm, FOV 400 × 290 mm, matrix 320 × 180 and ipat 2; (b) axial T_2_ mapping, TR 1000 ms, TE (18, 36, 54, 72, 90, 108), echo train 6, FOV 400 × 400 mm, matrix 256 × 256, slice thickness 3.5 mm and gap 28 mm. Acquisition time of the imaging sequence was 4 min. A parametric image was generated from T_2_ mapping sequence using Leonardo workstation (Siemens). Scout images and anatomical landmarks were obtained to ensure identical and time-efficient positioning in pre- and post- scans.

**Fig 2:**
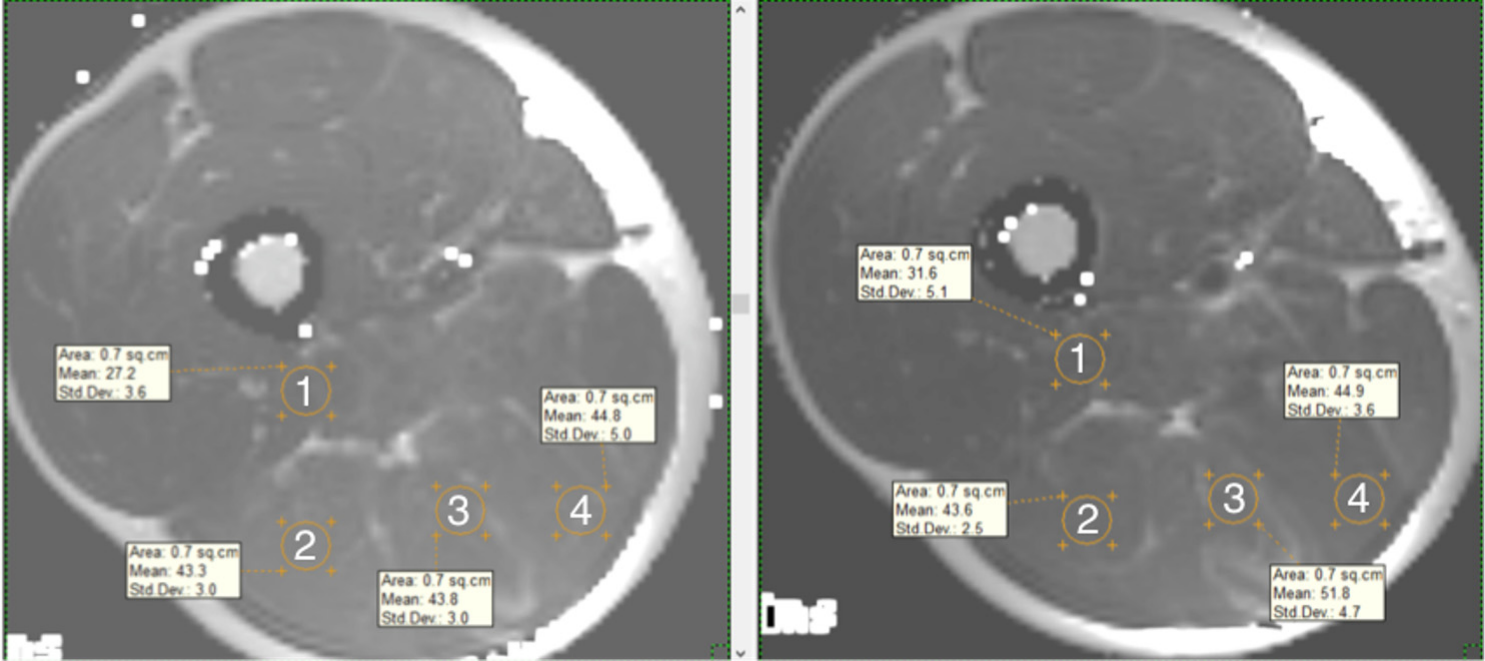
Selected MR images, obtained PRE and POST exercise, depicting regions of interest (ROIs). 1; m. biceps femoris short head, 2; m. biceps femoris long head, 3; m. semitendinosus, 4; m. semimembranosus.

T_2_ of muscles from the dominant leg were measured using eFilm Lite v.3.1 software (Merge Healthcare, Chicago, IL) [17]. Using the fat- suppressed images to detect any confounding artifact (e.g., vessels, fat), a circular region of interest (ROI) was selected for individual muscles (mm. BFl, BFs, SM, and ST) in each of the T_2_ mapping images where muscles were visible. Following pre-exercise scan analysis, the same-size circular ROI’s were placed in the T_2_ images of the post-exercise scan, to ensure identical positioning as in the pre-exercise analysis (Fig 2). A transverse (spin-spin) relaxation time measurement sequence with 3 TEs as applied to measure the absolute T_2_ value. Images taken at different TEs were fitted to a monoexponential time curve to extract the T_2_ values based on the formula: signal intensity = M0 x exp (-TE/T_2_), where signal intensity represents the signal intensity at a given TE and M0 is the original MRI signal intensity [17]. Site-specific muscle use was calculated after each exercise by obtaining the baseline and post-exercise average values of the first 30% axial scans where each muscle was visible starting from the hip/knee joint (proximal and distal portions, respectively) and middle scans (from 30% to 70%; mid portion) [13]. Two researchers, blinded to the origin of any image, independently analyzed all images. The intraclass correlation coefficients, coefficient of variation and typical error for the interrater agreement of the T_2_ values for the different muscle were: BFl (0.94, 2.4%, 0.95), BFs (0.99, 1.9%, 0.77), ST (0.99, 1.8%, 0.78) and SM (0.87, 4.0%, 1.60).

### Statistical analysis

Data in figures are presented as means ± standard deviation (SD) and coefficient of variation (CV) [(SD/mean x 100)]. All data were first log-transformed to reduce bias arising from non-uniformity error. Possible differences or changes in T_2_ values within- and between-muscle regions were analysed for practical significance using magnitude-based inferences by pre-specifying 0.2 between-subject SDs as the smallest worthwhile effect [22]. The standardized difference or effect size (ES, 90% confidence limit [90%CL]) in the selected variables was calculated. Threshold values for assessing magnitudes of the ES (changes as a fraction or multiple of baseline standard deviation) were >0.20, 0.20, 0.60, 1.2 and 2.0 for trivial, small, moderate, large and very large respectively (Hopkins et al. 2009). Quantitative chances of higher or lower changes were evaluated qualitatively as follows: <1%, almost certainly not; 1 −5%, very unlikely; 5−25%, unlikely; 25−75%, possible; 75−95%, likely; 95−99%, very likely; >99%, almost certain [22]. A substantial effect was set at >75% [23].

## Results

Players’ characteristics of each of the four groups were as follows: flywheel leg curl (age 19.2 ± 1.8 yr; height 180.1 ± 8.2; body mass 73.4 ± 9.6 kg), Nordic hamstring exercise (18.9 ± 1.7 yr; 176.8 ± 5.7 cm; 70.6 ± 3.8 kg), Russian belt deadlift (18.2 ± 1.4 yr; 173.9 ± 4.5 cm; 69.4 ± 5.2 kg) and hip extension kick conic-pulley (18.4 ± 1.2 yr; 179.1 ± 7.2 cm; 70.7 ± 5.0 kg). No substantial between group differences were observed.

Muscle use (i.e., pre and post measures) across the four differences exercises is displayed in Table 1.

**Table 1:** Site-specific T_2_ values of biceps femoris long head (BFl), biceps femoris short head (BFs), semitendinosus (ST) and semimembranosus (SM) before and immediately following four resistance exercises.

Exercise	Site-specific	BFl		BFs		ST		SM	
		Pre	Post	Pre	Post	Pre	Post	Pre	Post
Flywheel Leg Curl	Proximal	39.4 ± 6.2	42.6 ± 3.2	29.5 ± 2.9	50.4 ± 4.8	36.4 ± 4.7	59.0 ± 8.2	47.4 ± 6.1	49.7 ± 5.0
Flywheel Leg Curl	Medial	37.4 ± 3.5	43.3 ± 6.0	36.6 ± 5.1	53.9 ± 7.9	38.6 ± 2.8	65.0 ± 3.9	38.2 ± 3.5	41.4 ± 4.6
Flywheel Leg Curl	Distal	41.6 ± 2.4	48.8 ± 6.8	43.8 ± 3.1	61.7 ± 4.2	40.3 ± 3.3	65.0 ± 9.6	41.6 ± 2.4	48.8 ± 6.8
Nordic Hamstring	Proximal	40.9 ± 3.3	41.9 ± 4.2	28.2 ± 2.0	32.2 ± 3.3	36.1 ± 3.5	42.6 ± 5.8	44.2 ± 7.8	44.8 ± 7.3
Nordic Hamstring	Medial	37.4 ± 3.1	37.7 ± 3.8	40.4 ± 4.8	45.9 ± 5.3	39.3 ± 3.8	46.1 ± 5.5	38.8 ± 2.9	39.1 ± 3.5
Nordic Hamstring	Distal	41.4 ± 3.8	43.1 ± 4.1	43.0 ± 2.8	48.9 ± 6.2	39.3 ± 3.3	45.2 ± 4.7	42.2 ± 2.6	42.3 ± 2.5
Russian Belt Deadlift	Proximal	42.6 ± 2.5	45.6 ± 3.2	27.1 ± 3.0	28.7 ± 3.5	38.2 ± 4.3	42.0 ± 4.7	40.0 ± 2.8	44.1 ± 4.9
Russian Belt Deadlift	Medial	36.7 ± 3.8	39.3 ± 4.4	39.9 ± 2.5	40.7 ± 3.6	39.1 ± 3.5	43.4 ± 3.3	38.6 ± 2.2	41.4 ± 3.4
Russian Belt Deadlift	Distal	43.1 ± 1.9	45.7 ± 2.5	41.2 ± 3.4	44.3 ± 5.0	40.2 ± 1.6	43.6 ± 2.6	41.8 ± 2.0	44.3 ± 1.5
Hip extension conic-pulley	Proximal	40.8 ± 8.7	45.4 ± 9.3	28.9 ± 5.9	27.7 ± 3.1	35.6 ± 7.0	38.1 ± 7.0	39.7 ± 6.6	41.7 ± 7.9
Hip extension conic-pulley	Medial	37.7 ± 4.8	40.2 ± 4.0	38.9 ± 3.3	38.2 ± 3.4	36.8 ± 3.5	41.1 ± 3.4	38.8 ± 10.5	39.1 ± 9.0
Hip extension conic-pulley	Distal	42.8 ± 5.4	44.0 ± 4.7	41.1 ± 2.0	41.3 ± 2.1	39.0 ± 3.7	39.8 ± 3.8	41.5 ± 4.1	42.9 ± 4.8

**Fig 3:**
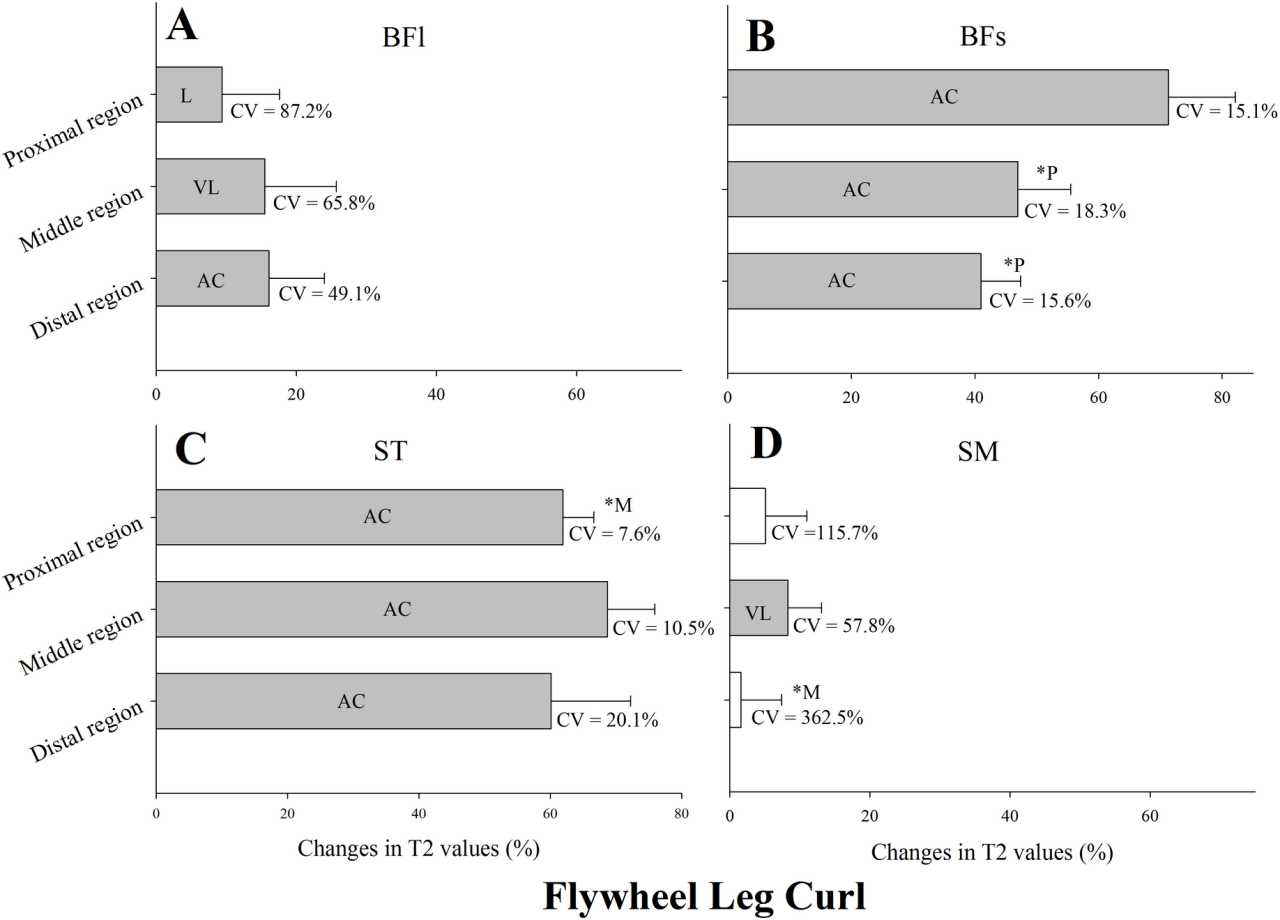
Mean, standard deviation and coefficient of variation (CV) of the change in the transverse relaxation time (T_2_) of the proximal, middle and distal regions of the biceps femoris long head muscle (BFl) and short head muscle (BFs), semitendinosus muscle (ST), and semimembranosus muscle (SM) immediately after four sets of eight repetitions of flywheel leg curl exercise. All values are given as a percentage of the pre-values. Closed bars represent substantial changes (L, likely; VL, very likely; AC, almost certain) while open bars display non-substantial changes. Asterisks indicate substantial differences between muscle regions.

Changes in T_2_ values after flywheel leg curl exercise are shown in Fig 3. T_2_ values were substantially higher following exercise in all regions of the BFl (Fig 3A), BFs (Fig 3B) and ST (Fig 3C) and middle region of the SM (Fig 3D). Changes in the proximal region of BFs were substantially higher than in the middle (ES = 2.50 ± 1.01) and distal (ES = 3.41 ± 1.18) regions (Fig 3B). T_2_ changes in the middle region of the ST were substantially higher than the changes in the proximal region (ES = 1.12 ± 0.83) (Fig 2C). CV of T_2_ changes in each muscle region are shown in Fig 3. The lowest CV was from a substantial change found in the proximal region of ST (CV = 7.6%), and the highest in the proximal region of BFl (CV = 87.2%).

**Fig 4:**
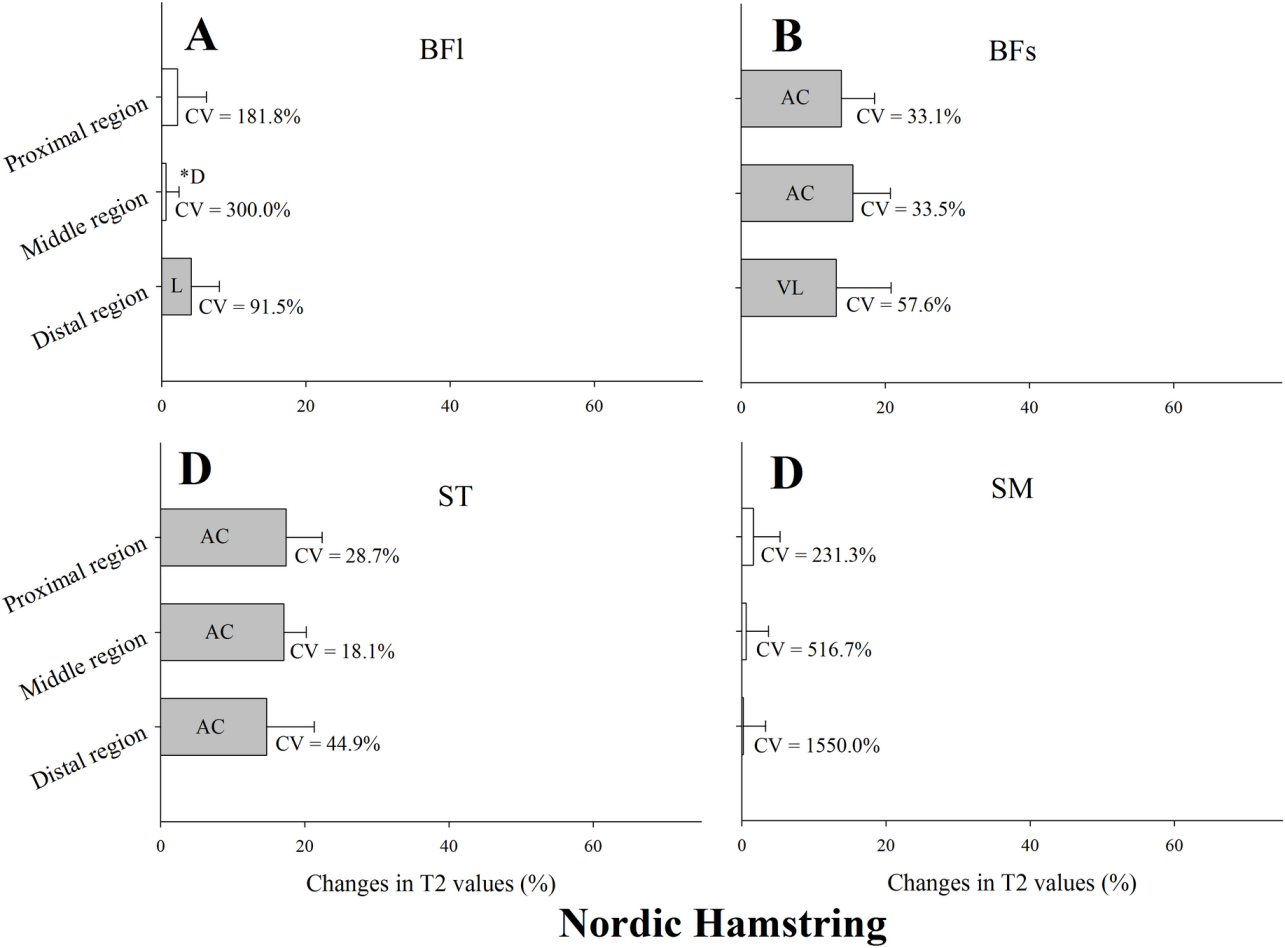
Mean, standard deviation and coefficient of variation (CV) of the change in the transverse relaxation time (T_2_) of the proximal, middle and distal regions of the biceps femoris long head muscle (BFl) and short head muscle (BFs), semitendinosus muscle (ST), and semimembranosus muscle (SM) immediately after four sets of eight repetitions of Nordic hamstring. All values are given as a percentage of the pre-values. Closed bars represent substantial changes (L, likely; VL, very likely; AC, almost certain) while open bars display non-substantial changes. Asterisks indicate substantial differences between muscle regions.

Changes in T_2_ values after the Nordic hamstring exercise are presented in Fig 4. T_2_ values were substantially higher following exercise in the proximal, middle and distal regions of the BFs and ST (Fig 4B and 4C, respectively). Only the distal region of the BFl displayed elevated T_2_ values (Fig 4A). No substantial T_2_ changes were observed in any region of the SM. CV of T_2_ changes in each muscle region are shown in Fig 4. The lowest CV from a substantial change was found in the middle region of ST (CV = 18.1%), and the highest in the distal region of BFl (CV = 91.5%).

**Fig 5:**
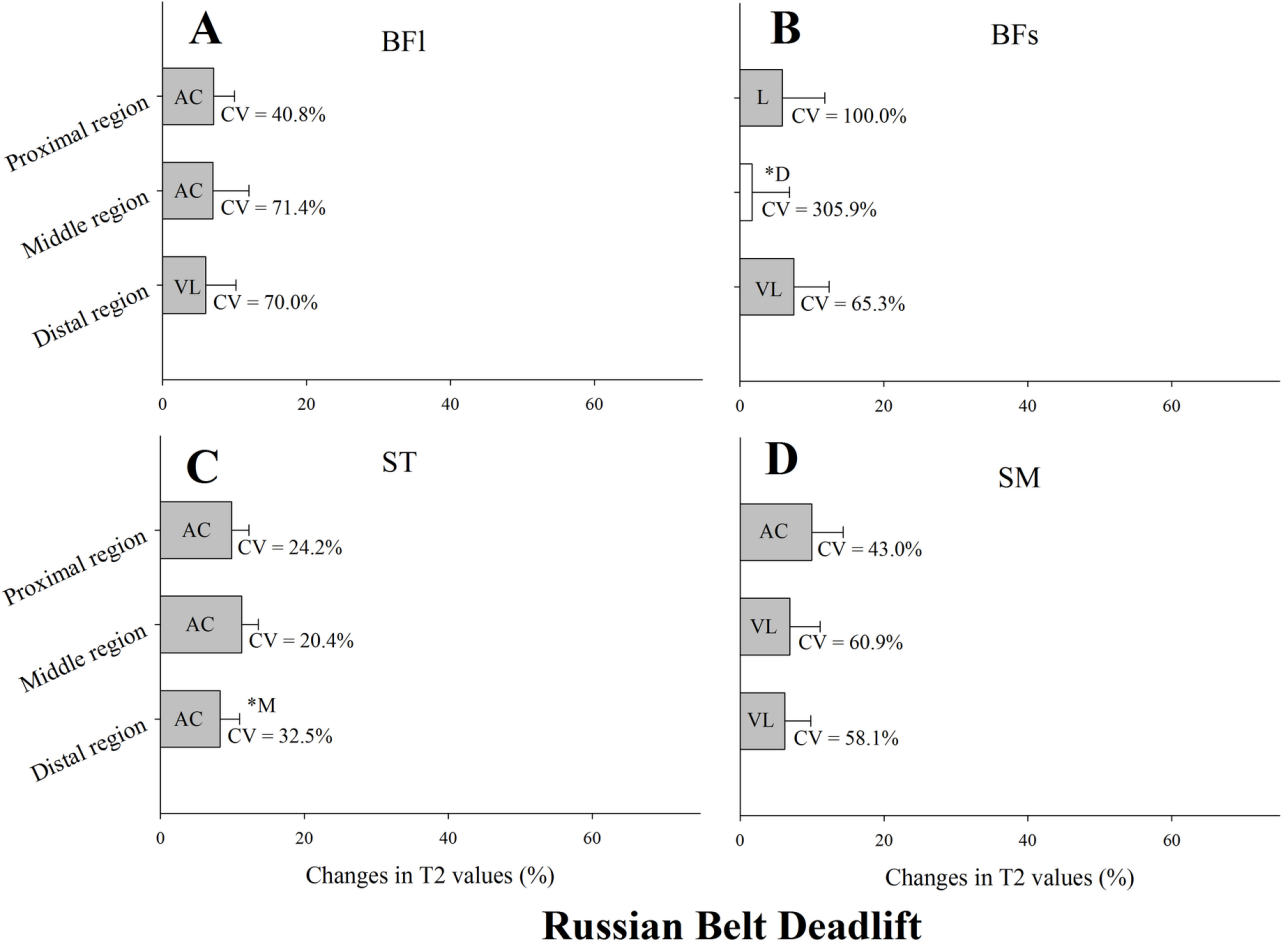
Mean, standard deviation and coefficient of variation (CV) of the change in the transverse relaxation time (T_2_) of the proximal, middle and distal regions of the biceps femoris long head muscle (BFl) and short head muscle (BFs), semitendinosus muscle (ST), and semimembranosus muscle (SM) immediately after four sets of eight repetitions of Russian belt deadlift. All values are given as a percentage of the pre-values. Closed bars represent substantial changes (L, likely; VL, very likely; AC, almost certain) while open bars display non-substantial changes. Asterisks indicate substantial differences between muscle regions.

Changes in T_2_ values after the Russian belt deadlift exercise are presented in Fig 5. T_2_ values were substantially elevated in all regions of the BFl, ST and SM (Fig 5A, 5C and 5D, respectively), while substantial changes in BFs were only observed in the proximal and distal regions (Fig 5B). Changes in the middle region of ST were substantially higher than the changes in the distal region (ES = 1.20 ± 0.84) (Fig 5C). CV of T_2_ changes in each muscle region are shown in Fig 5. The lowest CV from a substantial change was found in the middle region of ST (CV = 20.4%), and the highest in the proximal region of BFs (CV = 100.0%).

**Fig 6:**
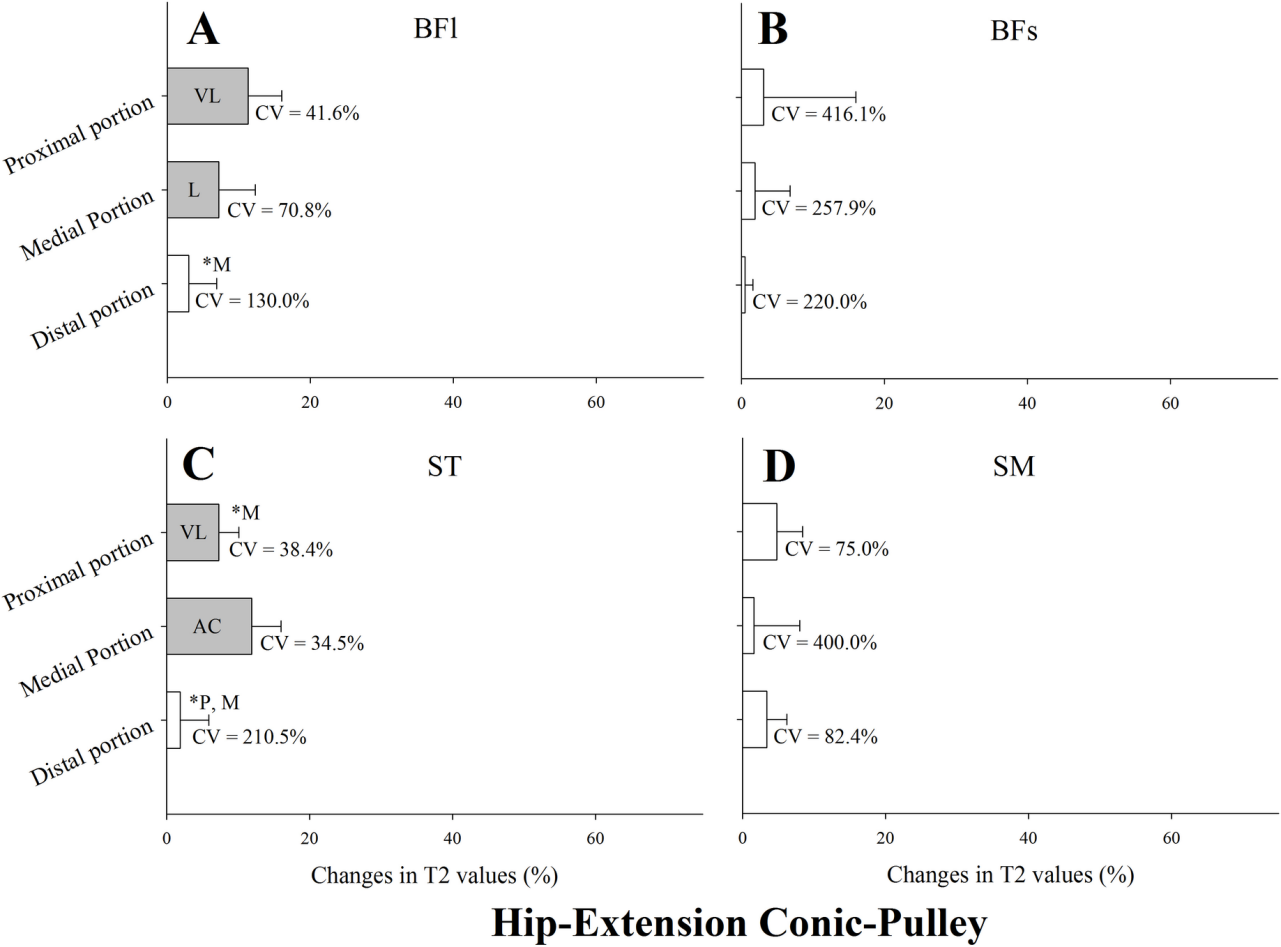
Mean, standard deviation and coefficient of variation (CV) of the change in the transverse relaxation time (T_2_) of the proximal, middle and distal regions of the biceps femoris long head muscle (BFl) and short head muscle (BFs), semitendinosus muscle (ST), and semimembranosus muscle (SM) immediately after four sets of eight repetitions of hip-extension conic-pulley. All values are given as a percentage of the pre-values. Closed bars represent substantial changes (L, likely; VL, very likely; AC, almost certain) while open bars display non-substantial changes. Asterisks indicate substantial differences between muscle regions.

Changes in T_2_ values after the hip extension kick conic-pulley exercise are presented in Fig 6. T_2_ values were substantially elevated in the proximal and middle regions of the BFl and ST (Fig 6A and 6C, respectively). There were no substantial changes in any region of the BFs and SM. Changes in the middle region of ST were substantially higher than in proximal (ES = 1.31 ± 0.85) and distal (ES = 2.47 ± 1.01) regions (Fig 6C). CV of T_2_ changes in each muscle region are shown in Fig 6. The lowest CV from a substantial change was found in the middle region of ST (CV = 34.5%), and the highest in the middle region of BFl (CV = 70.8%).

## Discussion

The hamstring muscles (ST, SM, BFl and BFs) are known to have different architectural and geometrical characteristics that translate into distinct inter- and intra-muscle functions during functional tasks such as resistance exercises calling for hip extension and/or knee flexion [13–16, 24]. The present study expands upon the existing knowledge by providing a comprehensive regional muscle use responses, assessed as the T_2_ of MR images, of the hamstring muscles during selected eccentric-oriented strengthening exercises, commonly used to prevent and/or rehabilitate hamstring injuries in elite soccer players. Mechanically, the four exercises evaluated in the present study are clearly different. Thus, rather to compare the four exercises, we will focus on the specific muscle use features of each exercise and how each exercise might be used for appropriated muscle conditioning in soccer players.

Observations of fMRI-derived non-uniform inter- and intra-muscle use during different hamstring strengthening exercises support preceding literature [13–16, 24]. Only the T_2_ regional muscle use of one of the four exercises investigated in the present study, the Nordic hamstring exercise, has been has been previously examined [14]. A non-uniform hamstring muscles response with intensified post-exercise T_2_ signal intensity in the ST and BFs muscles was reported [14]. While the authors reported some regional differences in MRI activity immediately after the exercise (e.g., greater T_2_ increase in the distal region of the BFs), our results showed a rather homogeneous regional muscle (i.e., ST and BFs) use. Moreover, in line with previous data [14, 17], we found a limited involvement of the BFl (Fig 4A). Despite the BFl contributes to knee flexion [13, 14], research has shown that changes in muscle (fascicle) length are more sensitive to hip movement compared to knee movement [25]. This difference might be related to the larger muscle moment arm at the hip resulting in greater excursion of the muscle with changing hip position [25]. Overall, the current study supports previous findings and suggests that the Nordic hamstring exercise can be recommended when the goal is to target ST and BFs.

The conventional leg curl weight-stack machine exercise is widely used in both soccer performance enhancement and injury prevention/rehabilitation settings. Previous studies have investigated the inter-muscle [16] and intra-muscular [13] regional differences in T_2_ changes after this exercise mode. Following a purely eccentric isotonic leg curl exercise [i.e., 120% of the 1 repetition maximum (RM)], Kubota et al. (2007) reported a significant muscle use increase of ST, BFs and to a lesser degree the BFl. Similarly, Ono et al. (2010) found a two- to three -fold greater ST compared to BFl muscle use immediately after a purely eccentric isotonic exercise at 120% of 1RM and isotonic combined concentric and eccentric exercise at 50% of 1RM. In the present study, despite the different contraction mode employed (i.e., inertial flywheel leg curl), the T_2_ increases in ST and BFs were also substantially higher than the observed in BFl in all the three muscle regions (4- to 6-fold and 2.5- to 11-fold, respectively, depending of the muscle region analyzed; Fig 2) [17]. Similar to the Nordic hamstring, the fact the hip position is fixed and unchanged during the execution of the exercise can limit the involvement of the BFl [25]. Indeed, the low to moderate (compared with the other CV values presented here) T_2_ between-player CV (7.6 to 20.1%) obtained (Fig 3B and 3C) seems to indicate quite homogenous individual responses in ST and BFs muscle use during the flywheel leg curl exercise. Of note is the relatively low CV (7.6%) observed in the proximal region of the ST, which in comparison with the CV observed in the same (proximal) muscle region of the BFl (87.1%), appears to confirm previous findings on the ST and BFs muscle dominance of the prone leg curl exercise [13, 16, 17]. In the present study, muscle use regional differences in ST and BFl were in line with previous data [13]; the proximal T_2_ changes of the ST were substantially lower than the middle with no substantial BFl between-region muscle use differences. On the contrary, Kubota and co-workers reported greater T_2_ changes in the BFs distal region [13] while in the present study BFs proximal T_2_ changes were substantially greater than both middle and distal regions. Overall, the flywheel leg curl exercise appears to impose a high, quite homogenous (i.e., all muscle regions) and reproducible (i.e., expected low between-players differences) ST and BFs muscle use in soccer players.

The stiff-leg deadlift and other deadlift variations are commonly used exercises aimed at developing hip extension strength and power. In addition, due to the hip and knee joint kinematics (i.e., large excursions combined with full knee extension), that are believed to impose a substantial lengthening of the hamstring muscle-tendon unit resulting in an eccentric overload at long muscle lengths [25], the inclusion of different deadlift variations have been recommended in several hamstring injury prevention and rehabilitation programs [26, 27]. In the current investigation, we employed a modified stiff-leg deadlift with the use of the “Russian belt” [17]. This device allows the player to bend forward in an explosive fashion, to emphasize the eccentric phase. While this is the first time that regional responses during the Russian belt deadlift are investigated, the global muscle use (i.e., fMRI) of the Russian belt deadlift has to date being examined only once, in the same group of trained soccer players [17]. Moreover, the global muscle use of a “classical” stiff-leg deadlift was previously assessed in a group of untrained subjects [24]. After 5 sets of 10 repetitions with a load of 60% of each subject body weight, T2 increased in SM (~12%), BFl (~8%) and to a lesser degree the ST (~5%). In the present study, T2 increases in the same range (4% to 12%) [24] were observed among all the regions of the ST, SM and BFl. Moreover, and in line with previous data [17, 24], the Russian belt deadlift was the only exercise examined here that displayed a substantial and consistent SM increases in T_2_ in all regions (Fig 5). Despite that the displayed T_2_ shifts are quite modest, it is worth noting that the Russian belt deadlift was performed with no additional external load but the player’s body weight. Moreover, the large hip excursion (i.e., hip flexion) due to the bending forward trunk movement, combined with a full knee extension is likely to result in the hamstring muscles undergoing a lengthening contraction, as both of these motions contribute to hamstring stretch [28, 29]. Albeit speculative, this substantial hamstrings lengthening might reduce muscle fiber work and being hamstring muscle-tendon unit compliance the main responsible for the force generation, which likely reduced muscle fiber work and metabolic energy expenditure [30].

The hip extension kick conic-pulley exercise is an open chain exercise, where by combining full knee extension with hip flexion-extension movements, a lengthening (eccentric) contraction under an inertial load (generated by the cone) is ensured. During this exercise, hip extensor muscles undergo fast stretch-shortening contractions (i.e., concentric hip extension is done during the descending phase and eccentric hip extension to counteract hip flexion is done during the recovering phase). Only the proximal and middle regions of the BFl and ST showed substantial increases in T_2_ while the other muscles and/or regions remained unchanged after the exercise (Fig 6). The ability of the hip extension kick conic pulley exercise to selectively recruit the proximal region of the BFl with a very limited involvement of other hamstring muscles may yield important consequences for current rehabilitation and injury prevention practices. The proximal region of the BFl has been reported to be the most frequently injured area in football players [4–7]. Moreover, BFl atrophy, often accompanied by hypertrophy of the BFs [31], and altered muscle architecture (i.e., shorter muscle fascicle and greater pennation angles) [32] have been observed in previously injured BFl compared to the uninjured contralateral limb. Thus, exercise interventions aimed at strengthening and reactivating the proximal BFl during eccentric contractions can benefit for the inclusion of the hip extension conic-pulley exercise.

### Limitations

Despite T_2_ relaxation time has been widely used for evaluating muscle recruitment during a wide range of exercises involving the hamstring muscles [13–15, 17], functional MRI has its own limitations. First, T_2_ changes do not reflect muscle use *per se*, but rather the metabolic response to muscle activation. Metabolic responses to strengthening exercises can vary greatly depending on the participation of the elastic structures, which can have a powerful effect on muscle force, power and work [30]. In this regard, work done by tendons and/or other elastic structures does not have to be performed by muscles; thus, tendons reduce muscle work and therefore metabolic cost, during stretch-shortening exercises [30]. Therefore, albeit speculative, the relatively modest changes in T_2_ values observed in some of the exercises investigated here (e.g., conic pulley exercise; S4 Video) might be related with the stretch-shortening behavior of the hamstring muscle complex as a result of the combined knee and hip joint kinematics, with the elastic series elements stretching to absorb the energy associated with decelerating the leg. Tendon compliance, therefore, could have acted as a mechanical buffer that reduces the stretch of muscle fibers and minimizes metabolic energy expenditure [30] Thus, in exercises in which the participation of tendinous tissues can substantially contribute to joint performance the muscle use information provided by the fMRI T_2_ changes, where only contractile tissue (i.e., muscle fibers) is examined, needs to be interpreted with caution. Future studies should quantify the possible roles that elastic structures might play in regulation force, power and work production during strength exercises. In addition, it should be acknowledged that high T2 vales (around 40 ms) were found at rest in the present study and elsewhere [13, 14, 16, 17], which could due to the imaging technique employed. However, as the outcome measure was the T2 changes (i.e., pre-post, within individual design), rather than absolute T2 values, our main conclusions would remain fundamentally unaltered.

### Perspectives

Different hamstring muscles and specific-regions within each muscle are likely to be selectively activated during different functional task that soccer players are required to do. Moreover, different muscles get injured at different locations, which might be the result of these differences in hamstring muscle involvement during soccer practices. Thus, knowledge of the differential muscle use of commonly employed strength exercises in soccer players appear relevant in deciding the exact strength exercises selection in order to prepare the player for a functional activity. From the results of this study and previous findings, it can be suggested that when the goal of the exercise intervention is to target the contractile elements of the BFs and ST and general hamstrings strengthening the flywheel leg curl and to a lesser extent the Nordic hamstring might be indicated. In contrast, for certain conditions that require the use of more functional, hip-dominant, stretch-shortening cycle muscle actions that promote the selective use of the proximal region of the BFl while minimizing recruitment of the other hamstring muscles, the hip extension conic-pulley would appear appropriate.

## Supporting Information

S1 Video Flywheel leg-curl exercise. (MP4)

S2 Video Nordic hamsting exercise. (MP4)

S3 Video Russian belt deadlift exercise. (MP4)

S4 Video Hip extension conic-pulley exercise. (MP4)
